# lnc001776 Affects CPB2 Toxin-Induced Excessive Injury of Porcine Intestinal Epithelial Cells via Activating JNK/NF-kB Pathway through ssc-let-7i-5p/*IL-6* Axis

**DOI:** 10.3390/cells12071036

**Published:** 2023-03-29

**Authors:** Kaihui Xie, Zunqiang Yan, Qiaoli Yang, Xiaoyu Huang, Pengfei Wang, Xiaoli Gao, Jie Li, Shuangbao Gun

**Affiliations:** 1 College of Animal Science and Technology, Gansu Agricultural University, Lanzhou 730070, China; 2 Gansu Research Center for Swine Production Engineering and Technology, Lanzhou 730070, China

**Keywords:** piglet diarrhea, lnc001776, ssc-let-7i-5p, CPB2 toxin, apoptosis, inflammatory injury, cell barrier

## Abstract

Piglet diarrhea caused by *Clostridium perfringens* (*C. perfringens*) type C (*CpC*) seriously endangers the development of the pig production industry. *C. perfringens* beta2 (CPB2) toxin is a virulent toxin produced by *CpC*. Long non-coding RNAs (lncRNAs) are key regulators in the immune inflammatory response to bacterial infection. Nevertheless, the functional mechanism of lncRNAs in bacterial piglet diarrhea is unclear. Herein, a novel lncRNA lnc001776 expression was confirmed to be substantially elevated in the ileum tissue of *CpC*-infected diarrhea piglets and in CPB2 toxin-treated porcine small intestinal epithelial cells (IPEC-J2). lnc001776 knockdown restrained CPB2 toxin-induced apoptosis, inflammatory injury, barrier dysfunction and activation of JNK/NF-kB pathway in IPEC-J2 cells. Additionally, ssc-let-7i-5p was identified as sponge for lnc001776. Overexpression of ssc-let-7i-5p repressed CPB2-induced injury in IPEC-J2 cells. Interleukin 6 (*IL-6*), a target gene of ssc-let-7i-5p, was enhanced in CPB2 toxin-treated IPEC-J2 cells. Rescue experiments demonstrated that a ssc-let-7i-5p mimic reversed the effect of lnc001776 overexpression on CPB2 toxin-induced IPEC-J2 cell injury and JNK/NF-kB pathway, whereas *IL-6* overexpression partially restored the impact of lnc001776. Overall, lnc001776 overexpression exacerbated CPB2 toxin-induced IPEC-J2 cell damage by sponging ssc-let-7i-5p to regulate *IL-6* to activate JNK/NF-kB pathway, indicating that lnc001776 could be a key target for piglet resistance to *CpC*-induced diarrhea.

## 1. Introduction

Piglet diarrhea is a typical disease in large-scale pig farms and a crucial cause of piglet death. It seriously affects the economic efficiency and development of pig farming. Piglet diarrhea is mainly caused by bacteria (*Salmonella*, *Escherichia coli* and *Clostridium perfringens* (*C. perfringens*)), viruses (Porcine Epizootic Diarrhea Virus (PEDV), Porcine Deltacoronavirus (PDCoV) and Porcine rotavirus (PoRV)) and parasites [1,2,3,4,5,6].

*C. perfringens* is a common pathogenic bacterium in nature, widely distributed in the intestinal tract of humans and animals. It can result in a diverse range of diseases, such as gas gangrene, necrotizing enterocolitis, animal enterotoxemia and human acute gastroenteritis [7]. The pathogenicity of *C. perfringens* is closely related to its secretion of more than 20 exotoxins, and different toxin types cause different diseases [8]. *C. perfringens* is classified into seven types (A–G), based on the six main toxins produced [9]. Among them, *C. perfringens* type C (*CpC*) causes necrotizing enterocolitis and enterotoxemia in lambs, calves and foals, especially in piglets [10]. Meanwhile, studies showed that *CpC* causes severe piglet diarrhea [3]. *CpC* produces two main types of exotoxins, α (CPA) and β (CPB), and *C*. *perfringens* beta2 (CPB2) exotoxin has been proven to be the main pathogenic toxin of *CpC* [11]. CPB2 toxin was isolated from piglets with *CpC*-infected necrotizing enteritis [12]. Previous studies indicated that CPB2 toxin had toxic effects on some intestinal cells [13]. Our studies found that recombinant CPB2 toxin caused inflammatory injury and apoptosis of IPEC-J2 cells and disrupted cell barrier function [14,15]. Therefore, studying the damaging impact of CPB2 toxin on IPEC-J2 cells may lay the foundation for the pathogenesis of *CpC*-infected piglet diarrhea.

Long non-coding RNAs (lncRNAs) are non-coding RNAs with more than 200 nucleotides in length [16]. lncRNAs are transcribed by RNA polymerase II and undergo 5′ splicing, capping and tailing to form a structure similar to that of classical mRNA, but with lower protein-coding capacity, higher tissue specificity and lower expression level than mRNA [17,18,19]. Prior studies have pointed out that lncRNAs have essential parts in diverse biological processes, including gene splicing, maintenance of stem cell properties, autophagy, cell proliferation, apoptosis and differentiation [20,21,22,23,24]. Furthermore, lncRNAs are closely associated with intestinal diseases caused by pathogenic microorganisms in livestock and poultry. Zhang et al. [25] found that lnc012227 bound to let-7g-5p, thereby indirectly regulating the expression mitogen-activated protein kinase kinase kinase 8 (*MAP3K8*) to regulate the infection of ducks against *Salmonella Enterica*. Wu et al. [2] demonstrated that lncRNA TCONS_00183659 was elevated in *Escherichia coli* F18-resistant piglets and bound to histone HistoneH4 to regulate myxovirus (influenza virus) resistance 1 (Mx1), Mx2 and interferon-induced protein with tetratricopeptide repeats 2 (IFIT2) protein expression to enhance the resistance of weaned piglets to *Escherichia coli* F18 infection. Chen et al. [26] identified TCONS_00223467 and TCONS_00241897 as candidate lncRNAs that participated in intestinal immunity between *Escherichia coli* F17 resistant and susceptible lambs. However, the role and potential mechanism of lncRNAs in *CpC*, causing piglet diarrhea, is unclear.

miRNAs are involved in a multitude of biological processes, mainly by repression of mRNA translation or enhancing its fragmentation [27]. Let-7 was found in *Caenorhabditis elegans* and is highly conserved and broadly expressed in the species, and is a very important gene regulator [28]. Let-7i-5p, a member of the let-7 family, has been found to play an essential role in cell proliferation, migration and invasion [29,30]. Additionally, let-7i-5p is associated with inflammatory damage processes and barrier function. For instance, Xiao et al. [31] found that let-7i-5p expression was markedly decreased in cycling exosomes and rostral ventrolateral medulla (RVLM), and that let-7i-5p attenuated the inflammatory response in pheochromocytoma cells (PC12). Harrington et al. [32] indicated that let-7i-5p was a carrier of endothelium-derived extracellular vesicles and was protective of the LPS-treated lung endothelial cell barrier. However, the potential mechanism of let-7i-5p in *CpC*-infected piglet diarrhea and intestinal inflammation needs further investigation.

In this study, we discovered that lnc001776 was up-regulated in the ileum tissue of *CpC*-infected diarrheic piglets, and its expression was elevated in CPB2 toxin-treated porcine small intestinal epithelial cells (IPEC-J2). lnc001776 knockdown ameliorated CPB2 toxin-induced apoptosis, inflammatory injury, barrier dysfunction and activation of JNK/NF-kB pathway in IPEC-J2 cells. Furthermore, lnc001776 promoted interleukin 6 (*IL-6*) expression through adsorption of ssc-let-7i-5p, thereby exacerbating CPB2 toxin-induced IPEC-J2 cell injury via JNK/NF-kB signaling pathway. This study suggests that lnc001776 could be a key regulatory molecule in the process of *CpC* infection in piglets.

## 2. Materials and Methods

### 2.1. Experimental Animal Handling and Sample Collection

In our previous study, 7-day-old Landrace × Yorkshire boars were instilled with a *CpC* culture (1 × 10^9^ CFU/mL) and control piglets (IC) were instilled with the same dose of culture, without the inoculated strain, to establish an animal model [33,34]. The top 5 piglets with the highest total diarrhea score were selected as the susceptible group (IS) and the top 5 with the lowest score were selected as the resistant group (IR). Ileum, jejunum, duodenum, lymph, spleen, kidney, liver, stomach, lung and heart tissues were collected from piglets in each group, quickly placed in liquid nitrogen and stored at −70 °C back in the laboratory. All animal experiments were guided by the Ethics Committee of the Experimental Animal Center of Gansu Agricultural University (Approval No. 2006-398).

### 2.2. Cell Lines Culture

The IPEC-J2 cells and human embryonic kidney 293T cells (HEK-293T) were available from BeNa Culture Collection (Beijing, China), cultured in dulbecco’s modified eagle medium (DMEM; HyClone, Logan, UT, USA) and supplemented with 10% fetal bovine serum (FBS; Gibco, Waltham, MA, USA) and 1% penicillin/streptomycin (Hyclone) at 37 °C, with 5% CO_2_ in a humidified atmosphere.

### 2.3. 5′ and 3′ Rapid Amplification of cDNA Ends (RACE)

The SMARTer^®^ RACE 5′/3′ Kit (TaKaRa, Dalian, China) was used to obtain the full-length sequence of lnc001776. The RNA was reverse transcribed to cDNA, as per the directions, and then 5′ and 3′ PCR amplification was carried out, respectively. The amplified products were recovered and purified and then ligated into the pMD19-T vector. Finally, sequencing was performed at Biotech Bioengineering Co., Ltd. (Shanghai, China). The primers were shown in Table S1.

### 2.4. Cytoplasmic and Nuclear RNA Fractionation Assay

The position of lnc001776 in the cytoplasm and nucleus in IPEC-J2 cells was detected by PARIS™ Kit (Invitrogen, Carlsbad, CA, USA). The 1 × 10^7^ IPEC-J2 cells were collected and separation experiments were performed, following the manufacturer’s specifications. A total of 500 μL pre-cooled Cell Fractionation Buffer reagent was added into cells, incubated on ice for 10 min and centrifuged at 1000 rpm at 4 °C for 5 min. Cytoplasmic RNA was present in the supernatant, and nuclear RNA was present in the precipitate. Supernatant was transferred to a new sterile centrifuge tube, 400 μL of pre-warmed 2 × Lysis/Binding Solution reagent was added, then 400 μL of anhydrous ethanol was added, centrifuged at 8000 rpm for 1 min and the filtrate discarded. Subsequently, 500 μL of Wash Solution 2/3 reagent was added and centrifugated at 4 °C for 1 min at 8000 rpm. Finally, 40 μL of pre-warmed Elution Solution reagent was added and centrifuged at 4 °C for 30 s at 8000 rpm, and the filtrate was retained for cytoplasmic RNA. The process of nuclear RNA extraction is the same as cytoplasmic RNA. The successfully separated cytoplasm and nucleus RNA were stored at −70 °C.

### 2.5. RNA-Fluorescence In Situ Hybridization (FISH) Assay

The subcellular localization of lnc001776 in IPEC-J2 cells was analyzed using a FISH kit (GenePharma, Shanghai, China). IPEC-J2 cells were placed in 24-well plates and the medium was discarded after the cells were plastered. Cells were fixed with 4% paraformaldehyde for 15 min. The following steps were then followed to perform the test: (a) Cells were treated with 200 μL of 0.1% Buffer A (PBS: Buffer A = 999:1) for 15 min. (b) Cells were incubated with 200 μL of 2 × Buffer C (ddH_2_O: 20 × Buffer C = 10:1) for 30 min at 37 °C. (c) lnc001776 probe mixture (Buffer E: lnc001776 probe = 100:1) was denatured at 73 °C for 5 min. (d) 100 μL of probe mixture was added to cells and hybridized overnight at 37 °C in an incubator. (e) The next day, cells were washed with 200 μL of 0.1% Buffer F (ddH_2_O: 20 × Buffer C: Buffer F = 750:150:1) and 200 μL of 2 × Buffer C for 5 min. (f) Cells were stained with DAPI solution and viewed with a fluorescence microscope (Olympus IX71, Tokyo, Japan).

### 2.6. Overexpression Plasmid Construction and Small Interfering RNA Synthesis

The sequences lnc001776 and *IL-6* (NM_001252429.1) CDS were amplified by PCR, employing specific primers containing restriction endonuclease sites of *Nhe* I and *Xho* I (TaKaRa). After PCR products were recovered and purified, they were cloned into pcDNA3.1(+) (Promega, Madison, WI, USA) vector to construct lnc001776 and *IL-6* overexpression plasmids. The successful overexpression vector was identified by sequencing and double digestion and named pc-lnc001776 and pc-IL-6. lnc001776 siRNA was designed by GenePharma Co., Ltd. lnc001776 siRNA and negative control (NC) sequences are summarized in Table S2.

### 2.7. Cell Transfection and CPB2 Toxin Infection

Our previous studies have described the process of CPB2 toxin purification and determined that 20 μg/mL of CPB2 toxin resulted in a 50% reduction in IPEC-J2 cell viability [14,15]. Therefore, in this study, we constructed a cell model by infecting IPEC-J2 cells with 20 μg/mL of CPB2 toxin.

pc-lnc001776, lnc001776 siRNA, ssc-let-7i-5p mimic, inhibitor, pc-IL-6 and their respective NCs were transfected into IPEC-J2 cells, exposed with CPB2-toxin, via Lipofectamine™2000 reagent (Invitrogen). The ssc-let-7i-5p mimic, inhibitor and their NCs sequences were generated by GenePharma Co., Ltd., and the sequences are presented in Table S2.

### 2.8. RNA Extraction and RT-qPCR

Total RNA was derived from IPEC-J2 cells and tissues via TRIzol reagent (Leagene, Beijing, China). RNA was reverse transcripted into complementary DNA (cDNA) by reverse transcription kits (Accurate Biotech, Changsha, China). Fast qPCR Mix (Meridian, Beijing, China) was used for RT-qPCR. The primers were composed by GENEWIZ Co., Ltd. (Suzhou, China). *β-actin* was thought as an inner reference. The gene levels were calculated through the 2^–(ΔΔCt)^ method [35]. The specific information on primers is summarized in Table S3.

### 2.9. Cell Viability Assay

Cell viability was estimated using the Cell Counting Kit-8 (CCK-8; absin, Shanghai, China). The IPEC-J2 cells at the logarithmic stage were placed in 96-well plates for transfection and CPB2 toxin treatment. After 24 h, 10 μL of CCK-8 reagent was added per well and fostered for 2 h. The absorbance was read at 450 nm through a multifunctional enzyme marker (Molecular Devices, Silicon Valley, CA, USA).

### 2.10. Proliferation Assay

5-ethynyl-2’-deoxyuridine (EdU) staining was performed to detect cell proliferation. Transfected and CPB2 toxin-treated IPEC-J2 cells were incubated with EdU working solution for 2 h at 37 °C. Subsequently, 200 μL of fixative solution was added per well, fixed at room temperature for 15 min and then stained, as described in the specifications of the EdU proliferation kit (Beyotime, Shanghai, China). Finally, the red proliferating cells were viewed under an inverted fluorescence microscope (Olympus IX71).

### 2.11. Apoptosis Analysis

Hoechst 33258 staining (Solarbio, Beijing, China) was administered to measure the apoptosis of IPEC-J2 cells. After treatment, 200 μL of Hoechst 33258 staining solution was added to each well of cells and left at room temperature for 5 min. The Hoechst 33258 staining solution was then aspirated and washed 3 times with PBS for 5 min each time. Then, it was viewed directly under a fluorescence microscope. (Olympus IX71).

### 2.12. Enzyme-Linked Immunosorbent Assay (ELISA)

ELISA kits (mlbio, Shanghai, China) were used to assess inflammatory cytokine levels. The cell supernatant of each group was collected into centrifuge tubes and centrifuged at 3000 rpm for 20 min. Then, the levels of inflammatory cytokines secreted by CPB2 toxin-infected IPEC-J2 cells were measured according to the reagent instructions for tumor necrosis factor-α (TNF-α), interleukin 1β (IL-1β), IL-8 and IL-10, respectively. Finally, the absorbance of each well was measured at 450 nm wavelength via a multifunctional enzyme marker (Molecular Devices).

### 2.13. Cytotoxicity Assay

Lactate dehydrogenase (LDH) activity assay kit (Solarbio) was used to measure the cytotoxicity of CPB2 toxin-infected IPEC-J2 cells. Cells were collected in centrifuge tubes; a total of 300 μL of extract was added to each tube and cells were broken up by ultrasound and centrifuged at 8000× *g* for 10 min at 4 °C. The supernatant was then transferred to a 96-well plate and spiked according to the instructions of the kits. After standing at room temperature for 3 min, the absorbance was measured at 450 nm on a multifunctional enzyme marker (Molecular Devices).

### 2.14. Reactive Oxygen (ROS) Level Assessing

ROS levels were detected by ROS Assay Kit (Beyotime). The cell culture medium was withdrawn and transfected, and CPB2 toxin-treated cells were incubated for 20 min at 37 °C with 350 μL of the fluorescent probe DCFH-DA. The cells were then washed three times with serum-free cell culture medium to remove any DCFH-DA that had not entered the cells. Finally, the intensity of fluorescence was measured at 488 nm excitation wavelength and 525 nm emission wavelength on a multifunctional enzyme marker (Molecular Devices).

### 2.15. Superoxide Dismutase (SOD) Activity Testing

SOD Assay Kit with WST-8 (Beyotime) was used to assess SOD activity in CPB2 toxin-infected IPEC-J2 cells. The cell culture was aspirated and washed once with pre-chilled PBS at 4 °C. A total of 200 μL of the SOD sample preparation was added to each well to fully lyse the cells. After centrifugation at 12,000× *g* for 5 min at 4 °C, the supernatant was transferred to a 96-well plate (20 μL/ well). Subsequently, 160 μL of WST-8/enzyme working solution and 20 μL of reaction starter working solution were added to each well, gently mixed and incubated for 30 min at 37 °C. Finally, the absorbance was measured at 450 nm on a multifunctional enzyme marker (Molecular Devices).

### 2.16. Dual Luciferase Reporter Assay

Partial sequences of lnc001776 and *IL-6* 3′UTR, containing a binding site to ssc-let-7i-5p, were amplified by PCR using specific primers containing *Xho* I and *Sal* I restriction endonuclease sites (TaKaRa). The amplification products were recovered and purified and ligated into the luciferase reporter vector pmirGLO (Promega) to construct lnc001776 and *IL-6* wild-type vectors (lnc001776-WT and *IL-6* 3′UTR-WT). The mutant vectors (lnc001776-Mut and *IL-6* 3′UTR-Mut) were synthesized by GENEWIZ Co., Ltd. Subsequently, ssc-let-7i-5p mimic and mimic NC were co-transfected in HEK-293T cells with lnc001776-WT, *IL-6* 3′UTR-WT, lnc001776-Mut and *IL-6* 3′UTR-Mut vectors, respectively. After 48 h of transfection, cells in each group were collected and then the fluorescence activity was detected, according to the instructions of Dual-Luciferase^®^ Reporter Gene Assay System (Promega). Finally, the ratio of firefly fluorescence activity to renilla fluorescence activity was used as a criterion to assess fluorescence activity.

### 2.17. Western Blot

The IPEC-J2 cells were cleaved in RIPA lysis buffer (Absin, Shanghai, China) to derive total protein. The BCA Protein Assay Kit (CretBiotech, Suzhou, China) was employed to determine protein concentration. After the denatured protein was processed by 10% SDS-PAGE electrophoresis, they were transferred to the PVDF membrane. The PVDF membrane was blocked with 5% skim milk for 1 h and then incubated with primary antibodies (Bioss, Beijing, China) at 4 °C overnight. The membrane was washed three times with TBST for 5 min and was hatched with goat anti-rabbit horseradish peroxidase (HRP) IgG antibody for 30 min at room temperature. The enhanced Chemiluminescence (ECL) Kit (NCM Biotech, Suzhou, China) was applied to visualize protein bands, and Image J software (National Institutes of Health, New York, NY, USA) analyzed gray levels. Specific information on the antibodies used in this research is exhibited in Table S4.

### 2.18. Statistical Analysis

The IBM SPSS 21.0 software (IBM Corp., Chicago, IL, USA) was employed to perform statistical analysis of the experimental data; two-way comparisons were performed using independent, sample t-test analysis and the values obtained were expressed as mean ± SD. * *p* < 0.05 was determined to be statistically significant, ** *p* < 0.01 was determined to be extremely statistically significant and ns indicated that the difference is not significant.

## 3. Results

### 3.1. lnc001776 was Highly Expressed in the Ileum of Diarrhea Piglets and CPB2-Exposed IPEC-J2 Cells

**Figure 1:**
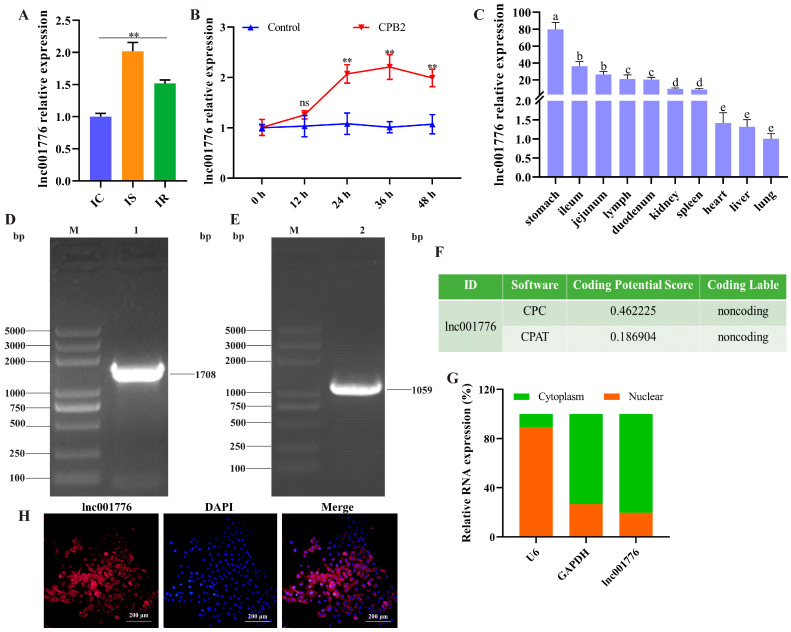
lnc001776 was elevated in CPB2 toxin-treated IPEC-J2 cells. (**A**) lnc001776 expression in IC, IS and IR groups by RT-qPCR. (**B**) lnc001776 expression in CPB2 toxin-infected IPEC-J2 cells by RT-qPCR. (**C**) lnc001776 levels in different tissues of healthy piglets via RT-qPCR. Different lowercase letters indicate highly significant differences between groups (*p* < 0.01). (**D**) 5′ RACE PCR amplification. (**E**) 3′ RACE PCR amplification. (**F**) lnc001776 coding ability prediction by CpC2 and CPAT. (**G**) lnc001776 expression in IPEC-J2 cytoplasm and nucleus. (**H**) lnc001776 location in IPEC-J2 cells through RNA-FISH. M: 5000 DNA marker; 1: 5′ RACE amplification product; 2: 3′ RACE amplification product. ns: not significant. ** *p* < 0.01.

In the previous high-throughput sequencing data from the ileum of *CpC*-infected diarrhea piglets, lnc001776 was found to be highly expressed in diarrhea piglets [36]. In this study, RT-qPCR results again confirmed that the lnc001776 level was obviously higher in the IS group than in the IC and IR groups (Figure 1A). Meanwhile, lnc001776 expression was markedly increased after 24 h of CPB2 toxin-exposed IPEC-J2 cells, and it peaked at 36 h (Figure 1B). Tissue expression profiles indicated that lnc001776 was most abundantly expressed in the stomach of healthy piglets and was moderately expressed in the intestinal tissues (Figure 1C). RACE experiment was performed to obtain the full-length sequence of lnc001776. 5′RACE and 3′RACE PCR amplification yielded 1708 bp and 1059 bp sequences, respectively (Figure 1D,E), and the full-length sequence of lnc001776 was obtained as 3386 bp after splicing and finishing. Subsequently, Coding Potential Calculator 2 (http://CpC2.gao-lab.org/, accessed on 7 November 2022 ) and Coding Potential Assessment Tool (https://wlcb.oit.uci.edu/cpat/, accessed on 7 November 2022) predicted that lnc001776 has no coding ability (Figure 1F). Cytoplasmic and nuclear RNA isolation and RNA-FISH assays results indicated that lnc001776 was localized in the IPEC-J2 cytoplasm (Figure 1G,H). These results imply that lnc001776 could be involved in *CpC*-infected piglet diarrhea through ceRNA regulatory mechanisms.

### 3.2. lnc001776 Deletion Suppressed CPB2 Toxin-Triggered Apoptosis in IPEC-J2 Cells

**Figure 2:**
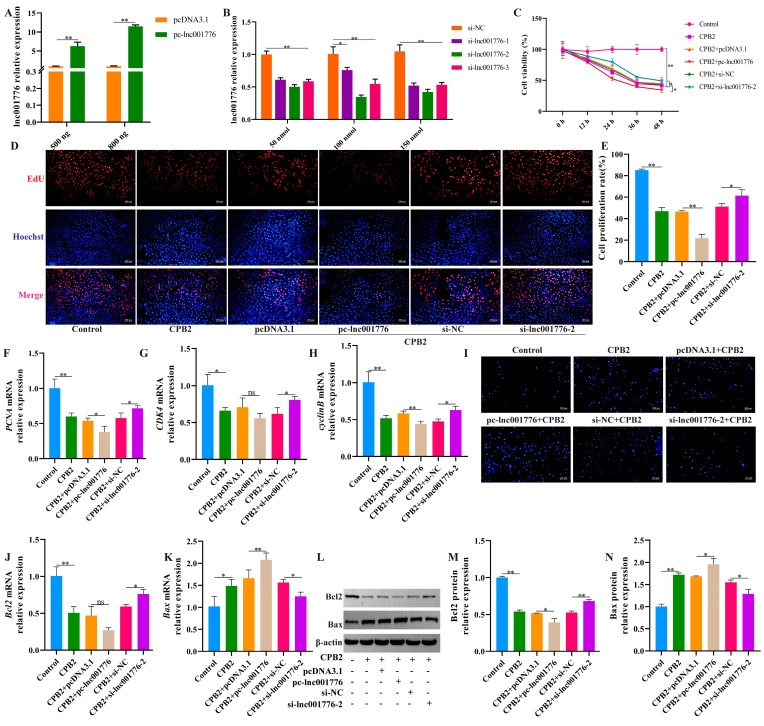
lnc001776 knockdown alleviated CPB2 toxin-triggered apoptosis in IPEC-J2 cells. (**A**) lnc001776 overexpression efficiency via RT-qPCR. (**B**) lnc001776 knockdown efficiency via RT-qPCR. (**C**) Cell viability through CCK-8. (**D**) Cell proliferating by EdU staining. (**E**) Percentage of red positive proliferating cells. (**F**–**H**) *PCNA*, *CDK4* and *cyclinB* levels through RT-qPCR. (**I**) Cell apoptosis by Hoechst 33258 staining. (**J**,**K**) Bcl2 and Bax mRNA expression via RT-qPCR. (**L**–**N**) Bcl2 and Bax protein expression through Western blot. ns: not significant. * *p* < 0.05, ** *p* < 0.01.

To determine the effect of lnc001776 on apoptosis, pc-lnc001776, si-lnc001776 and their respective NCs were transfected in CPB2 toxin-treated IPEC-J2 cells. pc-lnc001776 markedly elevated lnc001776 expression (800 ng; Figure 2A). Among the three si-lnc001776 siRNAs, si-lnc001776-2 had the most significant knockdown efficiency at 100 nmol (Figure 2B). CCK-8 assay and EdU staining results showed that lnc001776 silencing increased cell proliferation, while overexpression of lnc001776 showed the opposite results (Figure 2C–E). In addition, RT-qPCR results indicated that proliferating cell nuclear antigen (*PCNA*), cyclin-dependent kinase 4 (*CDK4*) and *cyclinB* levels were higher in the si-lnc001776-2 group than in the si-NC group (Figure 2F–H). Hoechst 33258 staining results demonstrated that lnc001776 knockdown dampened CPB2 toxin-generated apoptosis in IPEC-J2 cells (Figure 2I). Likewise, transfection with si-lnc001776-2 accelerated Bcl2 mRNA and protein levels (Figure 2J,L,M) and decreased Bax levels (Figure 2K,L,N). Overall, these data indicate that lnc001776 knockdown suppressed CPB2 toxin-generated apoptosis in IPEC-J2 cells.

### 3.3. Overexpression of lnc001776 Exacerbated CPB2 Toxin-Treated IPEC-J2 Cell Inflammatory Injury

**Figure 3:**
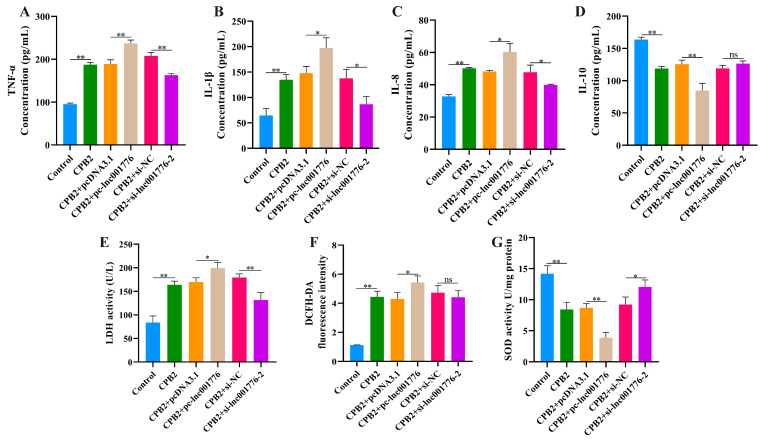
Overexpression of lnc001776 enhanced CPB2 toxin-triggered inflammatory damage in IPEC-J2 cells. (**A**–**D**) TNF-α, IL-1β, IL-8 and IL-10 levels via ELISA. (**E**) LDH activity. (**F**) ROS level. (**G**) SOD activity. ns: not significant. * *p* < 0.05, ** *p* < 0.01.

ELISA results demonstrated that elevated lnc001776 facilitated the release of TNF-α, IL-1β and IL-8 levels from CPB2 toxin-infected IPEC-J2 cells (Figure 3A–C) and inhibited IL-10 levels (Figure 3D). LDH is a cytoplasmic enzyme that is present in most cells. LDH activity changes when the cell membrane is damaged; therefore, the amount of LDH released can reflect the degree of cell damage [37,38]. Cytotoxicity assay results indicated that lnc001776 overexpression significantly increased LDH levels (Figure 3E). In addition, ROS levels and SOD viability were assayed to evaluate the effect of lnc001776 on CPB2 toxin-induced oxidative damage in IPEC-J2 cells. ROS is one of the main oxidants endogenously produced by cells and its transitional production causes oxidative stress in cells, damages intracellular biomolecules and induces apoptosis [39,40]. SOD, as the primary antioxidant for cells in vivo, is responsible for scavenging superoxide anions from the cytoplasm and mitochondria to protect cells from oxidative stress damage [41,42]. As displayed in Figure 3F,G, elevated lnc001776 stimulated ROS production (Figure 3F) and suppressed SOD activity (Figure 3G). These results suggest that elevated lnc001776 exacerbated the inflammatory damage induced by CPB2 toxin in IPEC-J2 cells.

### 3.4. Suppression of lnc001776 Mitigated CPB2 Toxin-Triggered Barrier Disruption in IPEC-J2 Cells

**Figure 4:**
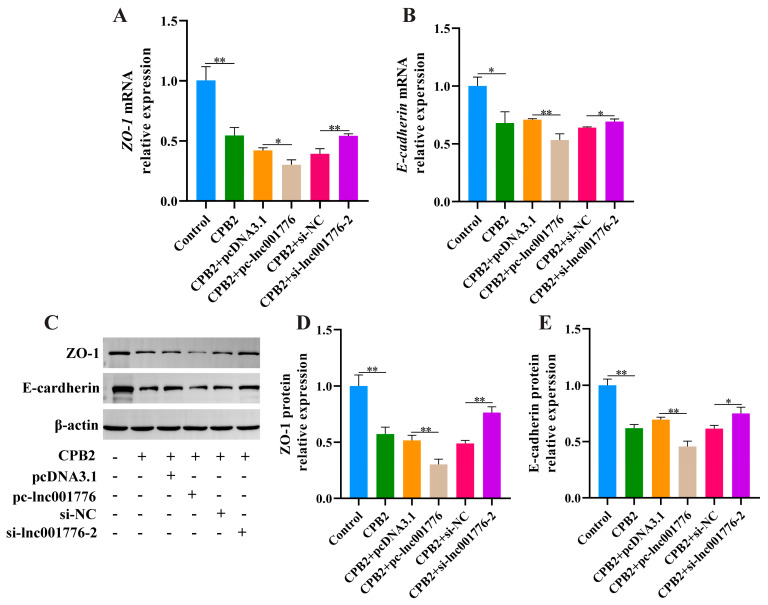
lnc001776 knockdown ameliorated the disruption of IPEC−J2 cell barrier integrity by CPB2 toxin. (**A**,**B**) ZO−1 and E-cadherin mRNA expression through RT-qPCR. (**C**–**E**) ZO−1 and E−cadherin protein expression via Western blot. * *p* < 0.05, ** *p* < 0.01.

We examined ZO-1 and E-cadherin expression to confirm the impact of lnc001776 on tight junctions of CPB2 toxin-treated IPEC-J2 cells. As depicted in Figure 4A–E, overexpression of lnc001776 repressed ZO-1, E-cadherin mRNA and protein expression, whereas lnc001776 knockdown enhanced their expression. These results reveal that inhibition of lnc001776 ameliorated the disruption of IPEC-J2 cell barrier integrity by CPB2 toxin.

### 3.5. lnc001776 Acted as a Sponge for ssc-let-7i-5p

**Figure 5:**
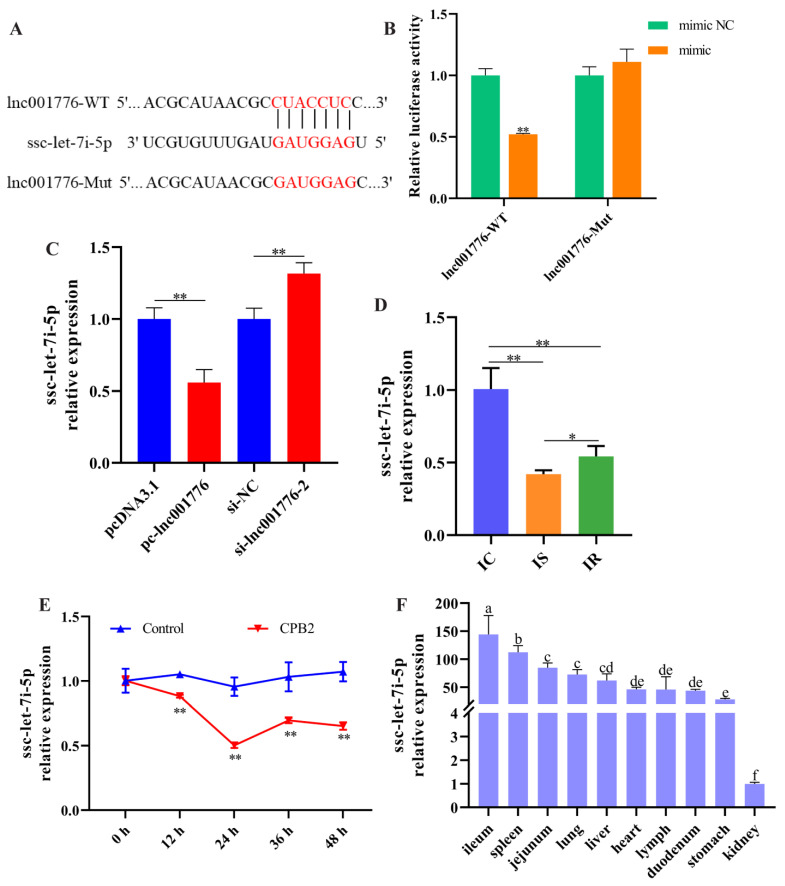
lnc001776 was a sponge for ssc-let-7i-5p. (**A**) Binding sites of lnc001776 to ssc-let-7i-5p. (**B**) Fluorescence activity through dual-luciferase reporter assay. (**C**) ssc-let-7i-5p expression after overexpression and knockdown of lnc001776 by RT-qPCR. (**D**) ssc-let-7i-5p expression in IC, IS and IR groups by RT-qPCR. (**E**) ssc-let-7i-5p expression in CPB2 toxin-infected IPEC-J2 cells via RT-qPCR. (**F**) ssc-let-7i-5p expression in different tissues of healthy piglets by RT-qPCR. Different lowercase letters indicate highly significant differences between groups (*p* < 0.01). * *p* < 0.05, ** *p* < 0.01.

We found a binding site for lnc001776 to ssc-let-7i-5p (Figure 5A), and dual-luciferase reporter assays indicated that ssc-let-7i-5p mimic remarkably reduced lnc001776-WT fluorescence activity, whereas it had no remarkable effect on lnc001776-Mut fluorescence activity (Figure 5B). Subsequently, RT-qPCR results showed that overexpression of lnc001776 suppressed ssc-let-7i-5p levels, and lnc001776 knockdown improved ssc-let-7i-5p levels (Figure 5C). Furthermore, ssc-let-7i-5p expression was remarkably lower in the IS group than in the IC and IR groups (Figure 5D). Similarly, ssc-let-7i-5p levels were reduced in CPB2 toxin-infected IPEC-J2 cells (Figure 5E), which was in contrast to lnc001776 expression, indicating a negative correlation between the two. Meanwhile, ssc-let-7i-5p was highly expressed in the ileum tissue of healthy piglets (Figure 5F). In conclusion, lnc001776 was a sponge for ssc-let-7i-5p.

### 3.6. Overexpression of ssc-let-7i-5p Ameliorated CPB2 Toxin-Caused Damage in IPEC-J2 Cells

**Figure 6:**
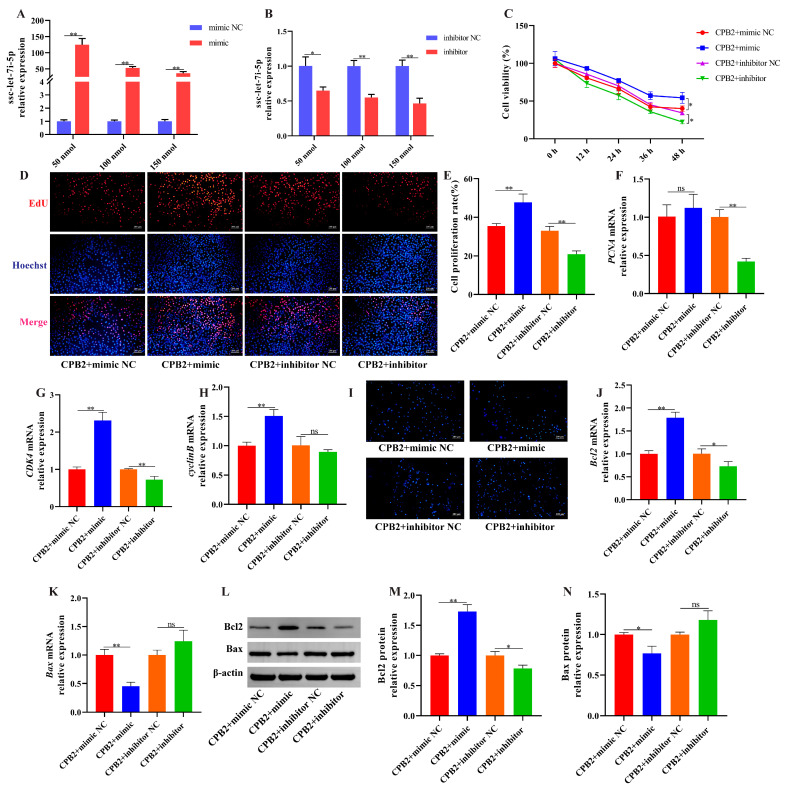
Overexpression of ssc-let-7i-5p alleviated CPB2 toxin-triggered apoptosis in IPEC-J2 cells. (**A**) ssc-let-7i-5p overexpression efficiency via RT-qPCR. (**B**) ssc-let-7i-5p knockdown efficiency via RT-qPCR. (**C**) Cell viability through CCK-8. (**D**) Cell proliferating by EdU staining. (**E**) Percentage of positive proliferating cells. (**F**–**H**) *PCNA*, *CDK4* and *cyclinB* levels through RT-qPCR. (**I**) Cell apoptosis by Hoechst 33258 staining. (**J**,**K**) Bcl2 and Bax mRNA expression via RT-qPCR. (**L**–**N**) Bcl2 and Bax protein expression through Western blot. ns: not significant. * *p* < 0.05, ** *p* < 0.01.

**Figure 7:**
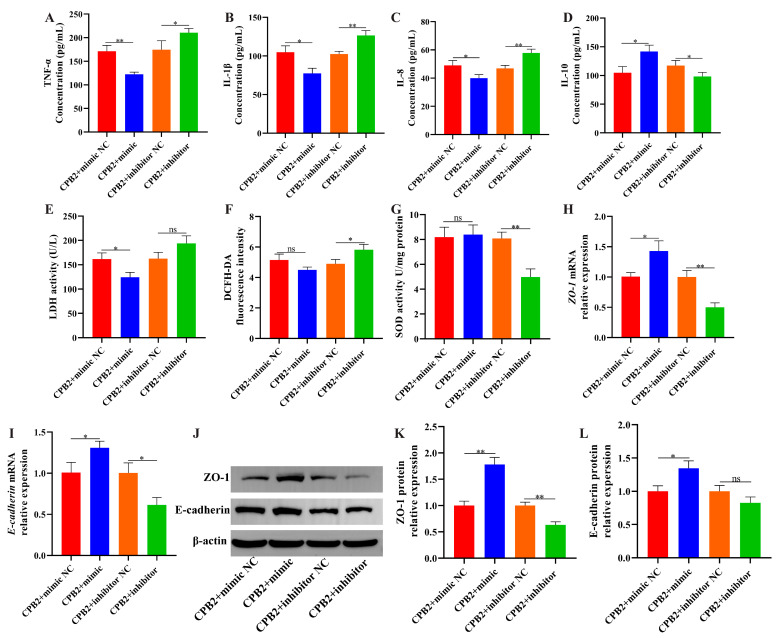
Elevated ssc-let-7i-5p ameliorated IPEC-J2 inflammatory damage and barrier dysfunction induced by CPB2 toxin. (**A**–**D**) TNF-α, IL-1β, IL-8 and IL-10 levels via ELISA. (**E**) LDH activity. (**F**) ROS level. (**G**) SOD activity. (**H**–**I**) ZO-1 and E-cadherin mRNA expression through RT-qPCR. (**J**–**L**) ZO-1 and E-cadherin protein expression through Western blot. ns: not significant. * *p* < 0.05, ** *p* < 0.01.

To investigate the role of ssc-let-7i-5p on CPB2 toxin-caused cell damage, we constructed a ssc-let-7i-5p mimic and inhibitor to overexpress and knock down ssc-let-7i-5p level in IPEC-J2 cells. As depicted in Figure 6A, the mimic significantly increased ssc-let-7i-5p expression (50 nmol). The inhibitor significantly decreased ssc-let-7i-5p expression (150 nmol; Figure 6B). Subsequently, CCK-8 assay and EdU staining indicated that ssc-let-7i-5p mimic enhanced cell proliferation (Figure 6C–E). Meanwhile, *PCNA* levels were not significantly changed after ssc-let-7i-5p overexpression (Figure 6F), and *CDK4* and *cyclinB* expression were elevated (Figure 6G,H). Hoechst 33258 staining revealed that ssc-let-7i-5p mimic inhibited apoptosis of damaged cells and the inhibitor demonstrated the opposite results (Figure 6I). In addition, Bcl2 levels were increased and Bax levels were decreased in ssc-let-7i-5p mimic group compared to the mimic NC group (Figure 6J–N). Additionally, transfection with ssc-let-7i-5p mimic significantly decreased TNF-α, IL-1β, IL-8 and LDH levels (Figure 7A–C,E) and increased IL-10 levels (Figure 7D). ssc-let-7i-5p overexpression had no significant influence on ROS contents and SOD activity, whereas ssc-let-7i-5p knockdown elevated ROS contents and decreased SOD activity (Figure 7F,G). As depicted in Figure 7H–L, high expression of ssc-let-7i-5p significantly increased ZO-1 and E-cadherin levels. Collectively, these results imply that overexpression of ssc-let-7i-5p suppressed CPB2-triggered IPEC-J2 cell injury.

### 3.7. IL-6 Was a Target of ssc-let-7i-5p

**Figure 8:**
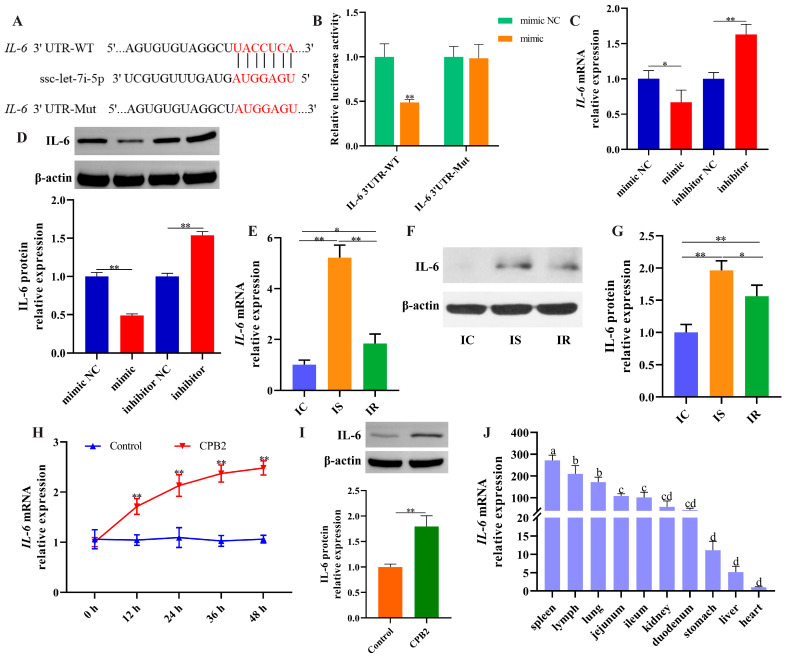
ssc-let-7i-5p directly targeted *IL-6.* (**A**) Binding sites of ssc-let-7i-5p to *IL-6*. (**B**) Fluorescence activity through dual-luciferase reporter assay. (**C**,**D**) IL-6 mRNA and protein expression after ssc-let-7i-5p overexpression and knockdown by RT-qPCR and Western blot. (**E**–**G**) IL-6 mRNA and protein expression in IC, IS and IR groups by RT-qPCR and Western blot. (**H**,**I**) IL-6 mRNA and protein expression in CPB2 toxin-infected IPEC-J2 cells via RT-qPCR and Western blot. (**J**) *IL-6* expression in different tissues of healthy piglets by RT-qPCR. Different lowercase letters indicate highly significant differences between groups (*p* < 0.01). * *p* < 0.05, ** *p* < 0.01.

TargetScan (https://www.targetscan.org/vert_72/, accessed on 20 February 2021) identified *IL-6* as a candidate target for ssc-let-7i-5p (Figure 8A). As depicted in Figure 8B, ssc-let-7i-5p mimic significantly diminished the fluorescence activity of IL-6 3′UTR-WT, whereas the fluorescence activity of IL-6 3′UTR-Mut was not notably changed (Figure 8B). *IL-6* levels were decreased after ssc-let-7i-5p overexpression and increased after ssc-let-7i-5p knockdown (Figure 8C,D). Additionally, *IL-6* expression was remarkably higher in the IS group than in the IC and IR groups (Figure 8E–G). Similarly, *IL-6* levels were notably increased in CPB2 toxin-treated IPEC-J2 cells after 12 h (Figure 8H,I). As shown in Figure 8J, *IL-6* expression was highest in the spleen of healthy piglets and moderately expressed in the intestinal tissues. These data demonstrate that ssc-let-7i-5p directly targeted *IL-6.*

### 3.8. Overexpression of lnc001776 Aggravated CPB2 Toxin-Triggered Damage in IPEC-J2 Cells by ssc-let-7i-5p/IL-6 Axis

**Figure 9:**
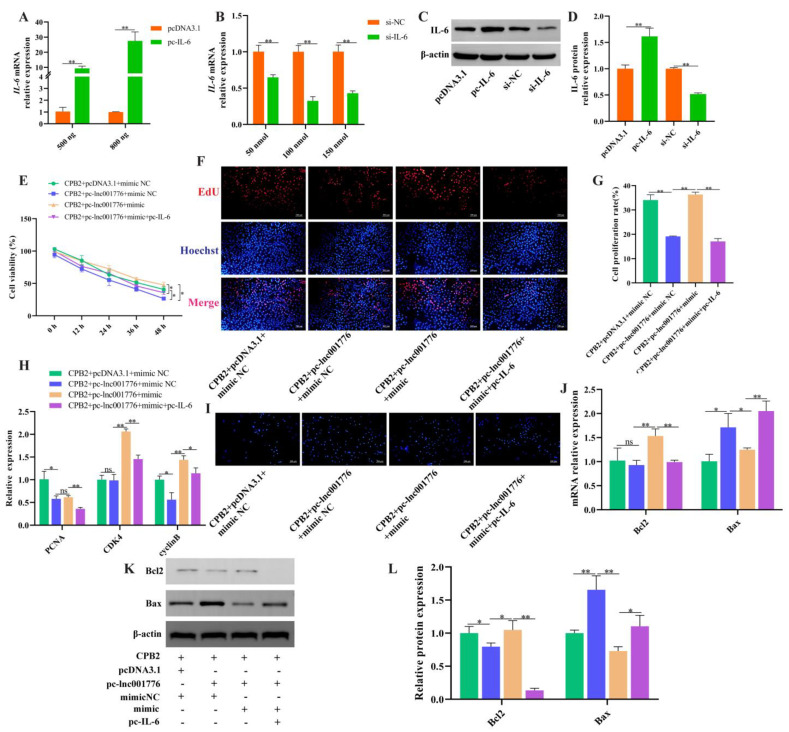
Overexpression of lnc001776 facilitated CPB2 toxin-induced apoptosis in IPEC-J2 cells by ssc-let-7i-5p/*IL-6* axis. (**A**–**D**) IL-6 overexpression and knockdown efficiency via RT-qPCR and Western blot. (**E**) Cell viability through CCK-8. (**F**) Cell proliferating by EdU staining. (**G**) Percentage of positive proliferating cells. (**H**) *PCNA*, *CDK4* and *cyclinB* levels through RT-qPCR. (**I**) Cell apoptosis by Hoechst 33258 staining. (**J**) Bcl2 and Bax mRNA expression via RT-qPCR. (**K**,**L**) Bcl2 and Bax protein expression through Western blot. ns: not significant. * *p* < 0.05, ** *p* < 0.01.

**Figure 10:**
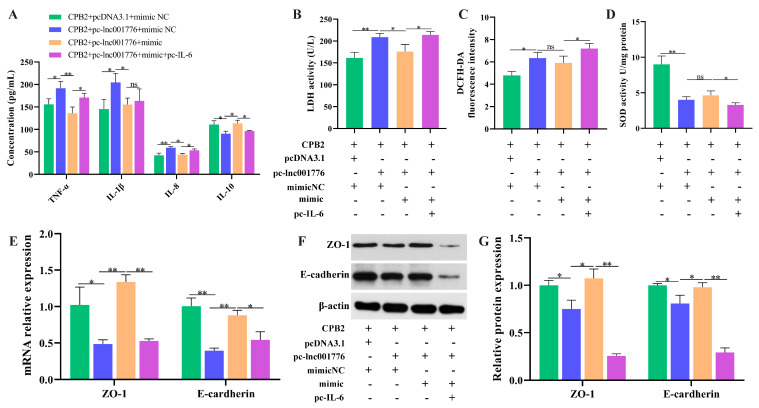
Elevated lnc001776 enhanced CPB2 toxin-triggered inflammatory injury and barrier dysfunction in IPEC-J2 cells via ssc-let-7i-5p/*IL-6* axis. (**A**) TNF-α, IL-1β, IL-8 and IL-10 levels via ELISA. (**B**) LDH activity. (**C**) ROS level. (**D**) SOD activity. (**E**) ZO-1 and E-cadherin mRNA expression through RT-qPCR. (**F**,**G**) ZO-1 and E-cadherin protein expression through Western blot. ns: not significant. * *p* < 0.05, ** *p* < 0.01.

Simultaneous overexpression of lnc001776, ssc-let-7i-5p and *IL-6* further verify that lnc001776 regulated CPB2 toxin-generated damage in IPEC-J2 cells by adsorption of ssc-let-7i-5p, to regulate *IL-6*. As shown in Figure 9A–D, IL-6 levels were successfully overexpressed and knocked down in IPEC-J2 cells. Overexpression of lnc001776 depressed cell proliferation, *PCNA* and *cyclinB* levels, as well as the introduction of ssc-let-7i-5p restored proliferation, *CDK4* and *cyclinB* levels, whereas elevated *IL-6* again performed suppression (Figure 9E–H). ssc-let-7i-5p mimic repressed lnc001776-induced apoptosis and Bax expression, while pc-IL-6 reversed this effect (Figure 9I–L). Transfection of pc-lnc001776 enhanced pro-inflammatory factors, LDH and ROS levels and decreased anti-inflammatory factors and SOD activity, while ssc-let-7i-5p reversed the effect of lnc001776 on inflammatory cytokines and LDH. Subsequently, pc-IL-6 rescued the effect of lnc001776 (Figure 10A–D). The disruption of cell barrier integrity by lnc001776 overexpression could be ameliorated by ssc-let-7i-5p. *IL-6* overexpression again underwent disruption (Figure 10E–G). Overall, overexpression of lnc001776 exacerbated CPB2 toxin-triggered IPEC-J2 cell injury via the ssc-let-7i-5p/*IL-6* axis.

### 3.9. Elevated lnc001776 Activated JNK/NF-kB Pathway Through ssc-let-7i-5p/IL-6 Axis

**Figure 11:**
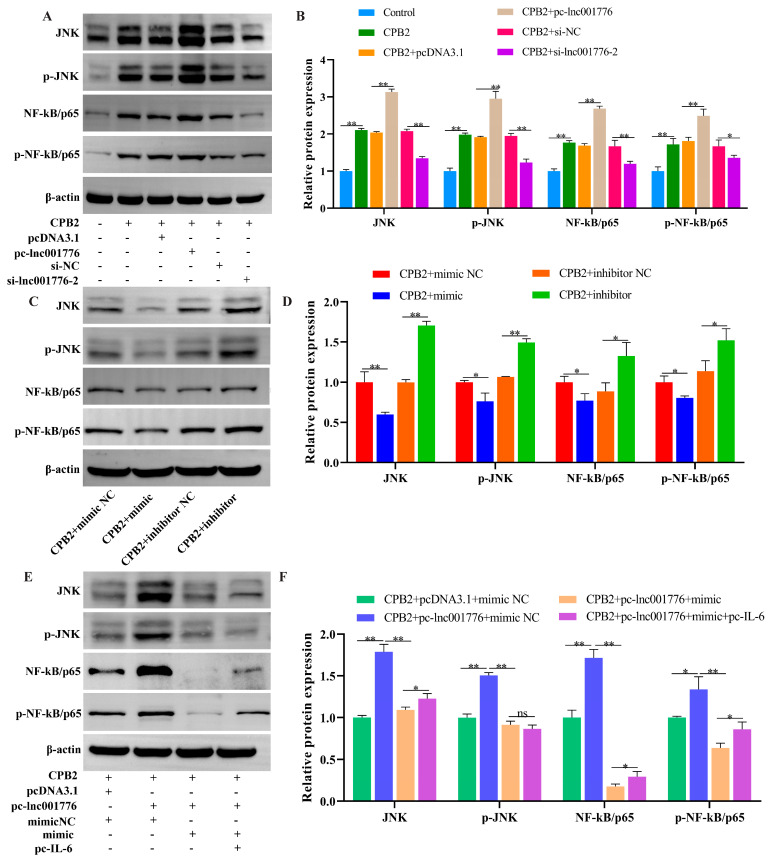
Overexpression of lnc001776 activated JNK/NF-kB pathway via ssc-let-7i-5p/*IL-6* axis. (**A**,**B**) JNK, p-JNK, NF-kB/p65 and p-NF-kB/p65 protein levels after overexpression and knockdown of lnc001776. (**C**,**D**) JNK, p-JNK, NF-kB/p65 and p-NF-kB/p65 protein levels after overexpression and knockdown of ssc-let-7i-5p. (**E**,**F**) JNK, p-JNK, NF-kB/p65 and p-NF-kB/p65 protein levels after simultaneous overexpression of lnc001776, ssc-let-7i-5p and *IL-6*. ns: not significant. * *p* < 0.05, ** *p* < 0.01.

The effect of lnc001776 on the JNK/NF-kB pathway was analyzed by Western blot. As displayed in Figure 11A–D, lnc001776 overexpression and ssc-let-7i-5p knockdown increased JNK, p-JNK, NF-kB/p65 and p-NF-kB/p65 protein levels in CPB2 toxin-induced IPEC-J2 cells, whereas lnc001776 knockdown and elevated ssc-let-7i-5p depressed their expression. Furthermore, ssc-let-7i-5p mimic weakened the promoting effect of lnc001776 on JNK, p-JNK, NF-kB/p65 and p-NF-kB/p65 protein levels, while the introduction of *IL-6* partially reversed the effect of let-7i-5p (Figure 11E,F). In conclusion, lnc001776 overexpression activated the JNK/NF-kB pathway via regulating the ssc-let-7i-5p/*IL-6* axis.

## 4. Discussion

Piglet diarrhea is a typical multi-factorial disease in large-scale pig farming, and *CpC* is one of the main bacteria causing piglet diarrhea and related intestinal inflammation, which seriously threatens the healthy development of the world pig industry [3]. Numerous studies demonstrated that lncRNAs play an essential role in host resistance to bacterial diarrhea. For instance, lncRNA FUT3-AS1 silencing enhanced resistance to *Escherichia coli* F18 in IPEC-J2 cells [43]. lncRNA IALNCR knockdown exerted antiviral functions through MAPK8/JNK1 pathway to promote Bovine viral diarrhea virus (BVDV) infection-generated apoptosis [44]. In this study, we confirmed that lnc001776 was elevated in *CpC*-infected diarrhea piglets and CPB2 toxin-exposed IPEC-J2 cells, demonstrating that lnc001776 may be involved in the process of *CpC*-infected piglet diarrhea. Several studies demonstrated that lncRNAs can participate in the immune response and defense process of intestinal inflammation and disease by regulating intestinal barrier function and mucosal immunity [45]. Wang et al. [46] found that lncRNA-CD244 was significantly up-regulated in CD8(+) T cells of mice infected with *Mycobacterium tuberculosis* and aggravated the inflammatory response. Elevated lncRNA GAS5 alleviated LPS-induced inflammatory injury and apoptosis in ATDC5 chondrocytes by regulating kruppel-like factor 2 (*KLF2*) [47]. Chen et al. [48] found that lncRNA H19 overexpression disrupted the partial function of the intestinal epithelial barrier. To verify the regulatory role of lnc001776 in CPB2-toxin-treated IPEC-J2 cells, we performed gain-of-function and loss-of-function experiments. The results showed that lnc001776 knockdown alleviated the apoptosis, inflammatory damage and barrier dysfunction of IPEC-J2 cells caused by CPB2 toxin, which further implies that lnc001776 plays a vital role in the resistance of piglets to *CpC*-infected diarrhea.

The regulation of lncRNAs varies depending on their subcellular localization. lncRNAs in the nucleus can exert transcriptional regulation, interacting with nuclear proteins and chromatin. However, lncRNAs in the cytoplasm are often able to exert post-transcriptional regulation, affecting mRNA stability or interfering with the translation process [49]. For instance, the lncRNA PYCARD-as1 recruited recombinant DNA methyltransferase 1 (DNMT1) and G9a to the PYCARD promoter to promote DNA methylation and modification of H3K9me2 in the nucleus, whereas lncRNA PYCARD-as1 could interact with PYCARD mRNA through 50 overlapping regions in the cytoplasm, thereby inhibiting ribosome formation in the cytoplasm for PYCARD translation [50]. An increasing amount of evidence indicated that lncRNAs can influence cellular functions by regulating mRNAs through adsorption of corresponding miRNAs [51]. lncRNA DANCR targeting miR-1306-5p/polo-like Kinase 1 (*PLK1*) axis reduced intestinal mucosal permeability and apoptosis of colonic mucosal epithelial cells and improved intestinal barrier dysfunction [52]. lncRNA TUG1 overexpression, targeting the miR-186-5p/X-linked inhibitor of apoptosis protein (*XIAP*) axis, attenuated NLRP3 inflammatory vesicle-induced cardiomyocyte scorching [53]. In the present study, lnc001776 was localized in the IPEC-J2 cytoplasm, suggesting lnc001776 could be involved in the regulation of *CpC*, causing piglet diarrhea, through a ceRNA mechanism. We found that lnc001776 acted as a sponge for ssc-let-7i-5p. lnc001776 overexpression suppressed ssc-let-7i-5p levels, whereas lnc001776 knockdown promoted ssc-let-7i-5p expression. Additionally, ssc-let-7i-5p was significantly down-regulated in *CpC*-infected diarrhea piglets and CPB2 toxin-exposed IPEC-J2 cells. Interestingly, we identified *IL-6* as a target of ssc-let-7i-5p. ssc-let-7i-5p mimic reduced *IL-6* levels and the inhibitor promoted *IL-6* levels. Furthermore, *IL-6* was enhanced in *CpC*-infected diarrhea piglets and CPB2 toxin-exposed IPEC-J2 cells. These findings suggest that lnc001776 could affect CPB2 toxin-induced damage in IPEC-J2 cells by regulating ssc-let-7i-5p/*IL-6* axis.

Several studies demonstrated that let-7i-5p is involved in inflammatory injury processes. For instance, let-7i-5p overexpression repressed hypoxia-induced NF-κB activation, mitochondrial dysfunction and cardiomyocyte apoptosis [54]. Let-7i-5p-loaded extracellular vesicles attenuated heme-induced inflammatory injury in heme-derived endothelial cells [55]. We found that elevated ssc-let-7i-5p inhibited apoptosis, inflammatory injury and barrier dysfunction in IPEC-J2 cells caused by CPB2 toxin. This study confirmed that lnc001776 is a damage factor in CPB2 toxin-exposed IPEC-J2 cells, while ssc-let-7i-5p is a protective miRNA. To further verify whether lnc001776 affects CPB2 toxin-generated damage in IPEC-J2 cells by regulating *IL-6* through adsorption of ssc-let-7i-5p, we overexpressed lnc001776, ssc-let-7i-5p and *IL-6* simultaneously. We found that ssc-let-7i-5p overexpression ameliorated the injury of IPEC-J2 cells by lnc001776, and *IL-6* overexpression weakened the protective effect of ssc-let-7i-5p on CPB2 toxin-induced IPEC-J2 cells. These data demonstrate that lnc001776 aggravated CPB2 toxin-generated injury in IPEC-J2 cells through ssc-let-7i-5p/*IL-6* axis.

**Figure 12:**
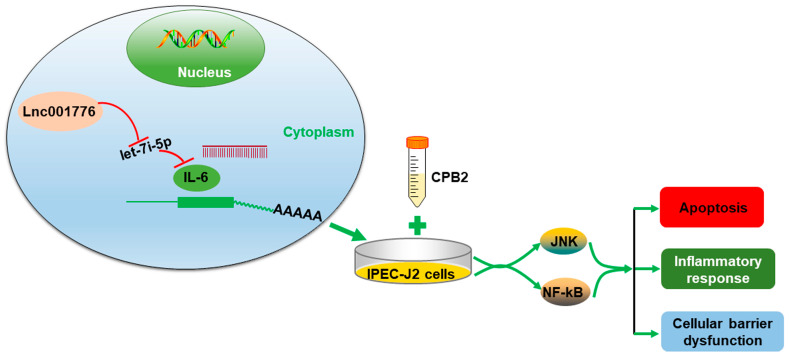
lnc001776/ ssc-let-7i-5p / *IL-6* regulatory axis regulates CPB2 toxin-induced IPEC-J2 cell damage through the JNK/NF-kB pathway. lnc001776 upregulates *IL-6* expression through adsorption of ssc-ssc-let-7i-5p, activates the JNK/NF-kB pathway, stimulates apoptosis and inflammatory responses, disrupts intestinal epithelial barrier function and, ultimately, exacerbates CPB2 toxin-induced IPEC-J2 cell damage.

The JNK pathway is one of the important pathways in the MAPK pathway that mediates immune signaling after pathogenic microbial infection and is involved in a variety of important physiological processes, such as host immune defense, cell proliferation, differentiation and inflammatory response [56]. NF-kB is one of the main pathways leading to the inflammatory response and activation of the NF-kB pathway, which triggers the generation of pro-inflammatory cytokines and exacerbates the inflammatory response [57,58]. We found that lnc001776 overexpression activated the JNK/NF-kB pathway, whereas ssc-let-7i-5p mimic inhibited the activation of the JNK/NF-kB pathway. Subsequently, *IL-6* overexpression alleviated the inhibition of the JNK/NF-kB pathway by ssc-let-7i-5p. Therefore, in this study, lnc001776 upregulated *IL-6* expression through adsorption of ssc-let-7i-5p and activated the JNK/NF-kB signaling pathway, which in turn exacerbated CPB2 toxin-induced IPEC-J2 cell injury (Figure 12).

## 5. Conclusions

In conclusion, lnc001776 was highly expressed in CPB2 toxin-induced IPEC-J2 cells and aggravated CPB2 toxin-induced IPEC-J2 cell injury by activating the JNK/ NF-kB pathway via ssc-let-7i-5p/*IL-6* axis, suggesting that lnc001776 could be a potential target for piglets to resist *CpC*-induced diarrhea.

## Data Availability

The original contributions presented in the study are included in the article/Supplementary Material. Further inquiries can be directed to the corresponding authors.

## Supplementary Materials

The following supporting information can be downloaded at: https://www.mdpi.com/article/10.3390/cells12071036/s1. Table S1: lnc001776 5′ and 3′ RACE amplification primer sequences; Table S2: si-lnc001776, ssc-let-7i-5p mimic, inhibitor, si-IL-6 and their NCs Sequences; Table S3: The RT-qPCR primer sequences used in this study; Table S4: Information on antibodies used in this study.
