# Supplementary material for: lnc001776 Affects CPB2 Toxin-Induced Excessive Injury of Porcine Intestinal Epithelial Cells via Activating JNK/NF-kB Pathway through ssc-let-7i-5p/IL-6 Axis
Source: Cells. 2023 Mar 29;12(7):1036. doi: 10.3390/cells12071036 (PMC10093645; doi:10.3390/cells12071036)
Supplement: Supplementary file 1 [file cells-12-01036-s001.zip › cells-2253133-SI.pdf]

Table S1. Lnc001776 5' and 3' RACE amplification primer sequences

Purpose	Sequences (5'-3')
5' RACE	Reverse1: GCACCACAGCCACAGCAGCACCAGAT
	Reverse2: GACCTACACCACAGCTCACAGCAACGTCA
3' RACE	Forward1: CCTTCTCTCTGAGACAGAACATCACAGCTA
	Forward2: CTGGAAGCCTCCAGGCATTCTTTACCCTTCA

Table S2. si-lnc001776, ssc-let-7i-5p mimic, inhibitor, si-IL-6, and their NCs Sequences

Name	Sense (5'-3')	Antisense (5'-3')
si-lnc001776-1	GGCUUGAGGUGCUCUUUAATT	UUAAAGAGCACCUCAAGCCTT
si-lnc001776-2	CGCACACACACCCAUAUAUTT	AUAUAUGGGUGUGUGUGCGTT
si-lnc001776-3	GAGGAGGCAAAUGAGAAAUTT	AUUUCUCAUUUGCCUCCUCTT
ssc-let-7i-5p mimic	UGAGGUAGUAGUUUGUGCU	CACAAACUACUACCUCAUU
si-NC/mimic NC	UUCUCCGAACGUGUCACGUTT	ACGUGACACGUUCGGAGAATT
ssc-let-7i-5p inhibitor	AGCACAAACUACUACCUCA	/
Inhibitor NC	CAGUACUUUUGUGUAGUACAA	/
si-IL-6	GGAUGAAGAACACAAAGAUTT	AUCUUUGUGUUCUUCAUCCTT

Table S3. The RT-qPCR primer sequences used in this study

Gene	Forward Sequences (5'-3')	Reverse Sequences (5'-3')	Accession No.
Lnc001776	TAACGCCTACCTCCCAGACA	GGCCTGTCCTGTTACCCAAA	/
IL-6	TCTGGTGATGGCTACTGCCT	CCGGAGAGGTGAAGAGCATTT	NM_001252429.1
Bcl2	CTTTGTGGAGCTGTATGGGC	GCCCGTGGACTTCACTTATG	XM_021099593.1
Bax	GGCCCTTTTGCTTCAGGGTTT	GACACTCGCTCAACTTCTTGG	XM_003127290.5
ZO-1	TGAGTTTGATAGTGGCGTTG	TGGGAGGATGCTGTTGTC	XM_021098827.1
E-cardherin	GAACCCACAGCCTCATGTCA	TCGGTCGTTGAACTCGATGG	EU805482.1
β-actin	ATATTGCTGCGCTCGTGGT	TAGGAGTCCTTCTGGCCCAT	XM_003124280.5
GAPDH	AGTATGATTCCACCCACGGC	TACGTAGCACCAGCATCACC	NM_001206359.1
ssc-let-7i-5p	TGAGGTAGTAGTTTGTGCT	mRQ 3' Primer (TaKaRa)	/
U6	GGAACGATACAGAGAAGATTAGC	TGGAACGCTTCACGAATTTGCG	NC_000015

Table S4. Information on antibodies used in this study

Protein	Item No.	Dilution proportion	Company
Bcl2	bs-4563R	1: 1500	Bioss
Bax	bs-0127R	1: 1500	Bioss
ZO-1	bs-1329R	1:1200	Bioss
E-cadherin	bs-1519R	1:1200	Bioss
IL-6	bs-4587M	1:1000	Bioss
JNK	GB13018-1	1:1000	Servicebio
p-JNK	GB13019-1	1:1000	Servicebio
NF-kB/p65	bs-0465R	1:1000	Bioss
p-NF-kB/p65	bs-3543R	1:1200	Bioss
β-actin	bs-0061R	1:2000	Bioss
Goat Anti-rabbit IgG/HRP	Bs-0295G-HRP	1:2000	Bioss
